# Optical Tunability and Characterization of Mg–Al, Mg–Ti, and Mg–Ni Alloy Hydrides for Dynamic Color Switching Devices

**DOI:** 10.1021/acsami.2c17264

**Published:** 2022-12-25

**Authors:** Kevin J. Palm, Micah E. Karahadian, Marina S. Leite, Jeremy N. Munday

**Affiliations:** † Department of Physics, University of Maryland, College Park, Maryland 20742, United States; ‡ Institute for Research in Electronics and Applied Physics, University of Maryland, College Park, Maryland 20742, United States; § Department of Electrical and Computer Engineering, University of California, Davis, California 95616, United States; ∥ Department of Materials Science and Engineering, University of California, Davis, California 95616, United States

**Keywords:** optical properties, alloys, thin film, Mg, metal hydride, switchable optical devices

## Abstract

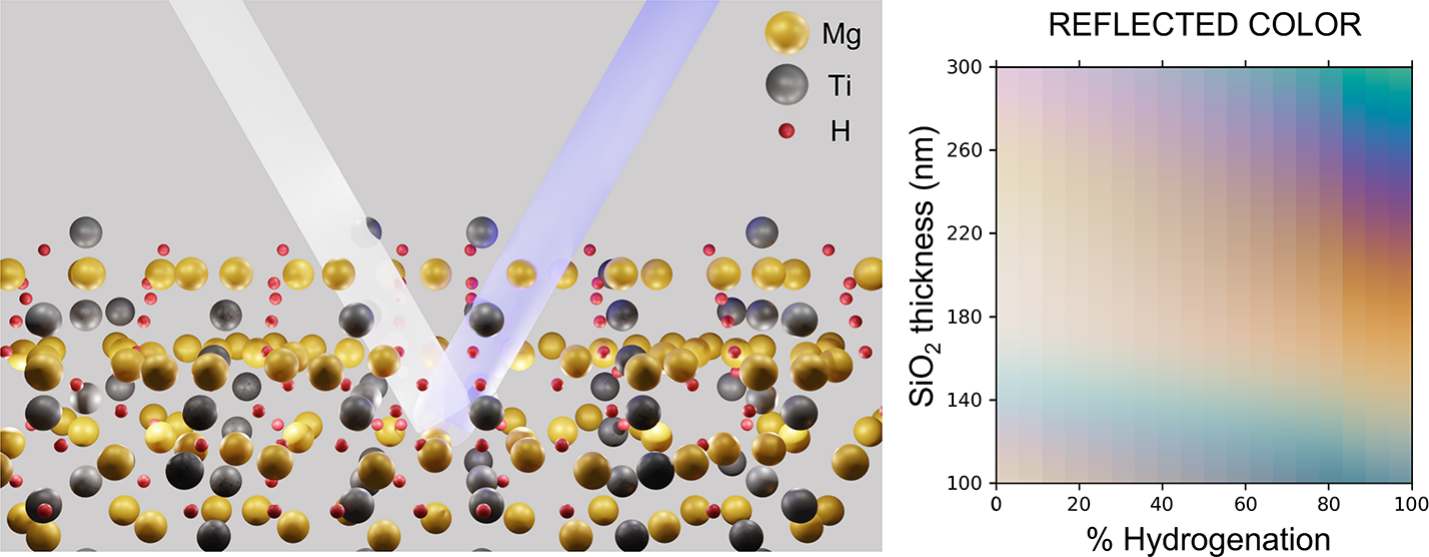

Mg shows great potential as a metal hydride for switchable optical response and hydrogen detection due to its ability to stably incorporate significant amounts of hydrogen into its lattice. However, this thermodynamic stability makes hydrogen removal difficult. By alloying Mg with secondary elements, the hydrogenation kinetics can be increased. Here, we report the dynamic optical, loading, and stress properties of three Mg alloy systems (Mg–Al, Mg–Ti, and Mg–Ni) and present several novel phenomena and three distinct device designs that can be achieved with them. We find that these materials all have large deviations in refractive index when exposed to H_2_ gas, with a wide range of potential properties in the hydride state. The magnitude and sign of the optical property change for each of the alloys are similar, but the differences have dramatic effects on device design. We show that Mg–Ti alloys perform well as both switchable windows and broadband switchable light absorbers, where Mg_0.87_Ti_0.13_ and Mg_0.85_Ti_0.15_ can achieve a 40% transmission change as a switchable window and a 55% absorption change as a switchable solar absorber. We also show how different alloys can be used for dynamically tunable color filters, where both the reflected and transmitted colors depend on the hydrogenation state. We demonstrate how small changes in the alloy composition (e.g., with Mg–Ni) can lead to dramatically different color responses upon hydrogenation (red-shifting vs blue-shifting of the resonance). Our results establish the potential for these Mg alloys in a variety of applications relating to hydrogen storage, detection, and optical devices, which are necessary for a future hydrogen economy.

## 1 Introduction

The chemical process of hydrogenation has been implemented as a mechanism to dynamically modify the optical properties of metallic thin films and nanostructures, ranging from pure elements to metallic compounds.^1−8^ Mg absorbs a large amount of hydrogen upon H_2_ exposure and goes through dramatic changes in its optical properties. One challenge of using pure Mg in dynamic metal hydride devices is that the hydride state is too thermodynamically stable and must be unloaded at a high temperature.^9^ The kinetics of both the absorption and the desorption are also slow, especially in bulk Mg, due to MgH_2_ being a poor proton conductor, which limits the diffusion of H through the material.^10^ In order to improve upon the kinetic and thermodynamic properties of Mg, alloying has been extensively used to destabilize the hydride phase and increase hydrogen diffusion. Alloying has been attempted with many metals including Co,^11−14^ Fe,^11,14−16^ and Mn,^14,17^ among others, but the most common secondary alloying elements used in the literature are Al,^18−22^ Ti,^7,23−27^ and Ni.^28−38^ These alloys have increased kinetics when compared to pure Mg^21,39−41^ and were each found to have interesting optical responses upon hydrogenation.

The Mg–Ni system in particular has been of interest in the optical community due to Mg_2_Ni forming an intermediate optical “black state” during its loading process.^33−35^ This black state is characterized by an intermediate loading state where the sample absorbs >75% of incident visible light when illuminated through a transparent substrate. The process has been investigated with ^15^N nuclear reaction analysis hydrogen depth profiling^35^ to determine the H atom vertical distribution in the sample. It was found that the black state formation is caused by preferential loading from the Mg_2_Ni/substrate interface, as opposed to the hydride being seeded near the dissociation sites at the Mg_2_Ni/Pd interface. A multi-layer interference effect occurs, where the bottom layer is fully hydrogenated Mg_2_NiH_*x*_ and the top layer remains metallic Mg_2_Ni. This layered orientation creates the observed black state. In the fully hydrogenated state, some Mg–Ni alloys have also been found to have good switchable window properties, switching from a reflecting state to a transparent state.^32^

The Mg–Ti system has been investigated for switchable solar absorbers,^25^ switchable mirrors,^42^ and hydrogen sensor applications.^43^ These materials have the advantage of having a highly absorbing state for wavelengths across the visible region for thick films (>200 nm) in their fully hydrogenated state, as opposed to Mg_2_Ni which obtains this highly absorbing state only during intermediate loading.^26^ This high-absorbing state occurs in Mg_*y*_Ti_1–*y*_ for *y* = 0.7–0.8. This alloying system is also of interest due to a decrease in degradation over many hydrogenation cycles, along with faster kinetics during these cycles, when compared to pure Mg.^26^ For very thin Mg–Ti films (<40 nm), the hydride state is more color-neutral than Mg–Ni alloys (which have a yellow tint), allowing for more aesthetically pleasing switchable windows, albeit with fairly low transmission amounts ∼20%.^42^

Mg–Al alloys are of interest for their high hydrogen weight percent (weight of hydrogen in a material divided by the total weight of the material) for hydrogen storage along with much faster hydrogenation kinetics at room temperature when compared to Mg.^22^ These materials also have been suggested as switchable window devices as Mg–Al alloys with ∼70% Mg have been demonstrated to have color-neutral transmission.^21^

To date, all optical measurements reported in the literature of these alloys consist of using normal incidence reflection and transmission measurements to determine their properties, such as the absorption coefficient and/or the optical band gap (e.g., in Mg–Ti^26^ and Mg–Ni alloys^31,44^) and the dielectric function by of the reflection and transmission data with a Drude–Lorentz model (e.g., in Mg–Ni alloys^35,45^). These methods of obtaining thin-film optical properties are much less sensitive than variable-angle spectroscopic ellipsometry, especially for very thin films <50 nm.^46^ There have been no reports in the literature of the optical properties of Mg–Al alloys, or of any Mg–Ti, Mg–Ni, or Mg–Al alloys in intermediate loading states. Our system allows for in situ ellipsometry to dynamically investigate the optical properties of thin films (<50 nm) of these materials with high sensitivity as they are hydrogenated.^1,2,47^ By using similar deposition parameters to fabricate the different alloy systems, these measurements also allow us to quantitatively compare the responses of these different systems.

In this work, we measure in situ the dynamic optical properties of different atomic ratios of thin-film Mg–Al, Mg–Ti, and Mg–Ni alloys as they are exposed to H_2_ gas. We also quantify the loading and stress values of these alloys during their exposure to H_2_. We find large optical changes for all the alloys investigated, with Mg_0.85_Ti_0.15_ exhibiting the largest optical changes for any alloy. We confirm the optical black state in the Mg–Ni samples through transfer-matrix method (TMM) simulations using our measured optical properties and observe the highest absorbing state for Mg_0.73_Ni_0.27_, as expected with that atomic composition being the closest to Mg_2_Ni. We demonstrate three potential dynamic devices: switchable windows, switchable light absorbers, and tunable color filters. The Mg–Ti alloys exhibit the best properties for each application, with Mg–Ni alloys showing the widest range of different property changes depending on the alloy composition. Overall, our in situ experiments, quantitative analysis, and computational designs lay the foundation for scalable and robust devices. Our material selection and their intrinsic optical behavior are well-suited for photonic devices where reversible color changes are required.

## 2 Experimental Section

Each thin-film sample is fabricated by room-temperature physical vapor deposition cosputtering. Two separate AT-cut 5 MHz quartz crystal microbalances (QCMs), a glass slide, and a Si chip are included in the deposition chamber for each deposition. Prior to deposition, samples are cleaned with acetone, methanol, isopropanol, and water. The alloys are deposited through a 12.5 mm diameter shadow mask centered on the QCM substrate. The direct current powers of the sputtering tool ranged from 50 to 450 W to attain the different alloy compositions. These minimum and maximum values are determined by the minimum voltage necessary to maintain a plasma and the voltage limit of the tool, respectively. Each sample was capped with a 3 nm Pd layer without breaking vacuum to catalyze the hydrogenation reaction and prevent surface oxidation of the sample. The composition of each alloy is determined with EDX taken on the Si chip included in the deposition chamber, taking an average of five measurements at different points on the sample to ensure uniform alloying.

The optical properties of the materials are determined with in situ spectroscopic ellipsometry using the system described in Palm et al.^1^ To determine the thickness of the Mg alloy films, the glass slide sample that is included in the deposition chamber is measured with ellipsometry and transmission measurements. The raw Ψ and Δ data are then fit with an optical model with the properties of the Mg alloy and the thickness of that alloy as fit parameters. The optical properties of the glass substrate are taken before the metal deposition and are defined in the model. The properties of the sputtered Pd are measured separately and are defined in the model. The thickness of the Pd layer is set to be 3 nm in the model for all alloy samples. With the transmission measurements, this fitting procedure allows for unambiguous determination of the thickness of the Mg alloy films. The thicknesses of the films we investigated ranged from 19 to 42 nm. As a consistency check for our thickness measurements, we use the same model properties and thicknesses on the data taken on the Au QCM electrode substrate and find good agreement without refitting the model parameters.

The volume expansion of these Mg-based alloys is an important factor in the thin-film optical model fittings. The Mg alloy thin films investigated here have appreciable transmission, and the optical effect of the substrate must be taken into account. For these alloys, the volume expansion for each is found to be ∼15% over the atomic ratio region that we are investigating,^35,48^ and we use this value for all of our samples. To include this expansion in the model, we use the dynamic loading data to determine how much H is in the film at each optical time step and then scale the total thickness expansion by the same ratio as the current loading to the final loading value (i.e., if a film’s final calculated loading value is 1 and at time step *t*, we calculate that the loading is H/M = 0.33, then we define the volume expansion at this time step to be 0.33 × 15% = 5%). H/M is defined to be the number of hydrogen atoms per metal atom in the metal alloy lattice, with the total number of metal atoms equaling the number of Mg atoms added to the number of atoms from the secondary alloying element. We also define the loading amount of the Pd capping layer by this same ratio. At each time step, with the thicknesses and Pd cap properties defined, we then use a B-spline model with 0.3 eV node spacing to fit the optical properties of the Mg-alloy.

The hydrogen loading for each alloy is measured on the second QCM sample with the method outlined in a study by Murray et al.^47^ To convert from the QCM frequency to the H/M loading ratio, the density of each sample must be input into the calculation. For Mg–Ti, both Mg and Ti have hexagonal close-packed lattice structures, and no known intermetallic states form. Because of this behavior, we believe that a linear weighting of the densities of Mg and Ti in the same ratio as their atomic percent in the alloy is a reasonable approximation of the density. As a check of this assumption, we compare densities calculated from linear weighting with densities calculated from lattice constant measurements using X-ray diffraction measurements^23^ and find good agreement (<6% difference). For the Mg–Al system, we also choose to use this linear weighting scheme because there are no other alloy phases found for this material when the atomic Mg percent >60%.^21^ For the Mg–Ni system, we use a slightly different method. Mg_2_Ni is a known intermetallic with a density of 3.48 g/cm^3^ and because this forms a separate phase within the Mg–Ni alloys, a linear combination of pure metal densities is not applicable. From ref (44), we know for Mg_*y*_Ni_1–*y*_ if 0.67 < *y* < 0.89, the alloy forms a varying mixture of crystalline Mg_2_Ni and amorphous Mg_0.89_Ni_0.11_, but the lattice constant remains constant in this region. If 0.89 < *y* < 0.95, the lattice constant begins to expand, and the alloy is mainly nanocrystalline Mg_2_Ni and crystalline Mg. Using this knowledge of the phases, we use the following equation to calculate the densities of the Mg–Ni alloys for 0.67 < *y* < 0.89
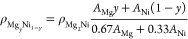
1where ρ_Mg_2_Ni_ is the density of Mg_2_Ni, *A*_Mg_ is the atomic mass of Mg, and *A*_Ni_ is the atomic mass of Ni. Here, we calculate the density by multiplying the mass ratio of the alloy to Mg_2_Ni with the known density of Mg_2_Ni. This result is using the fact that the volume of the lattice is not changing from the Mg_2_Ni size, thus allowing us to only account for mass. For higher Mg percent with 0.89 < *y* < 0.95, we have
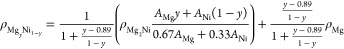
2where ρ_Mg_ is the density of pure Mg. Here, we linearly weight the density of Mg_0.89_Ni_0.11_ (calculated with eq 1) with the density of Mg. This rationale uses the fact that for samples with up to 89% Mg, there is no expansion in the lattice, and with additional Mg added above the 89% point, we are adding crystalline Mg with the density ρ_Mg_. Using these calculated densities and the thicknesses found from the optical fittings, we can then calculate the loadings and stresses with the method outlined in a study by Murray et al.^47^

## 3 Results and Discussion

### 3.1 Optical Properties of Mg Alloy Hydrides

In the following sections, we report and discuss the dynamic optical properties of our fabricated Mg–Al, Mg–Ti, and Mg–Ni alloys as they are exposed to 1 atm H_2_ gas.

#### 3.1.1 Mg–Al Hydrides

**Figure 1:**
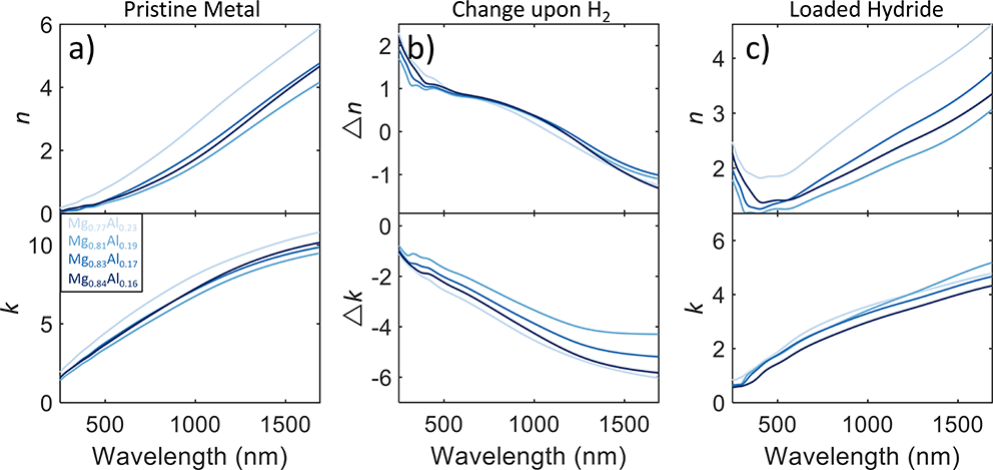
Optical properties *ñ* = *n* + i*k* of four different Mg–Al alloy hydrides. (a) Optical properties in the pristine metallic state before hydrogenation. (b) Change in optical properties upon hydrogenation, defined here as the pure metal optical properties subtracted from the hydride optical properties. (c) Optical properties in the fully hydrogenated hydride state. Each colored line on the plot represents a different atomic ratio of metal hydride, with darker shades representing higher atomic Mg percent ratios.

Figure 1 shows the measured optical properties *ñ* = *n* + i*k* of four different compositions of Mg–Al alloys and how these properties change under complete hydrogenation under 1 bar H_2_ pressure. For these materials, we observe a small spread in the initial and final properties of the materials, which is as expected due to all the alloys being close together in the atomic composition. The reason for this proximity in composition is a relative lack of sensitivity of the different materials to different deposition voltages (i.e., 200 W Mg and 200 W Al powers compared to 450 W Mg and 50 W Al powers only give a 7% difference in atomic Mg percent). We find that both the real and imaginary parts of the index of refraction increase with longer wavelengths for the alloy in the metallic state as we expect from most lossy metals. Interestingly, we find that metallic Mg_0.77_Al_0.23_ exhibits the largest *n* and *k* across the spectrum, as well as the largest optical change in *k*. We find that for all the Mg–Al alloys, the change in the optical properties upon hydrogenation follow the same trend. For Δ*n*, there is little variation from sample to sample. Each sample exhibits a positive Δ*n* for shorter wavelengths and a negative change for longer wavelengths, with the crossover point from positive to negative Δ*n* occurring between 1100 and 1225 nm. All samples exhibit a decrease in *k* across the measured spectrum, with the largest decreases occurring in the NIR and the smallest decreases in the visible spectrum. Despite having the largest Δ*k*, fully loaded Mg_0.77_Al_0.23_H_*x*_ still has the largest *k* across the visible spectrum, although only by a small margin, and the second highest in the NIR. In the final hydride state, as shown in Figure 1c, we find that each of the materials still has significant attenuation in the long-wavelength visible and into the NIR spectrum, with *k* > 3 after ∼1000 nm for each alloy. This behavior is despite the fact that the NIR optical properties saw the largest decreases in *k*. For the real part of the index of refraction, we find a minimum in the mid-visible spectrum that increases to large values (>3) in the NIR. The hydride samples are still somewhat optically metallic, not exhibiting a complete transition to a dielectric material.

**Figure 2:**
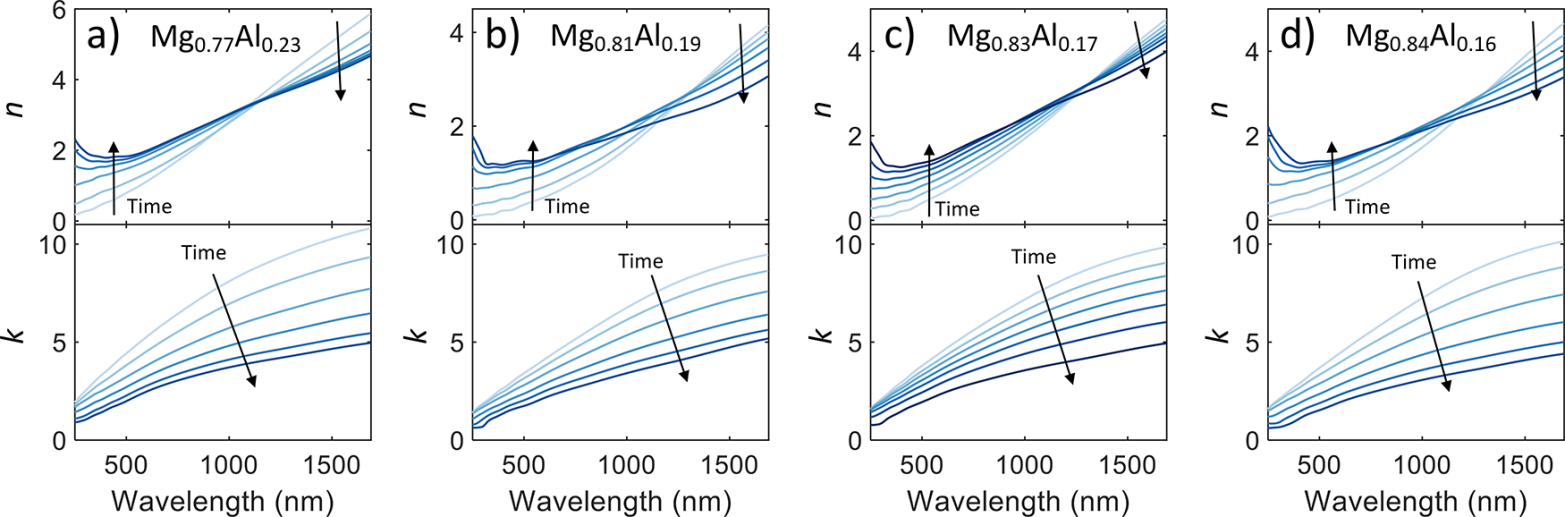
Optical properties of different Mg–Al alloys as they are exposed to H_2_ gas. The lightest colored line depicts the alloy in the pristine metallic state. As H_2_ is introduced to the system, the material begins to hydrogenate, denoted by the lines getting darker in the plot, with the darkest line indicating the full hydride state. Each line is not linearly spaced in time and is instead chosen to aesthetically show the range of possible intermediate states. The alloys shown here are (a) Mg_0.77_Al_0.23_, (b) Mg_0.81_Al_0.19_, (c) Mg_0.83_Al_0.17_, and (d) Mg_0.84_Al_0.16_.

As shown in Figure 2, we observe the intermediate states during the hydrogen loading. These plots show a smooth transition of the optical properties from the metallic (lighter colored curves) to the hydride states (darker colored curves). Note that the chosen optical curves on this plot are not linearly spaced with time but are instead chosen to show the range of properties of the intermediate states. Loading generally begins slowly as small amounts of H_2_ are introduced to the chamber, then increases quickly during the beginning of the α to β phase transition, and finally slows down to a long tail for the final ∼10% of the load when most of the material is in the β phase. Most of our materials show similar time dynamics, with the total time of loading ranging from 10–15 min for most samples.

#### 3.1.2 Mg–Ti Hydrides

**Figure 3:**
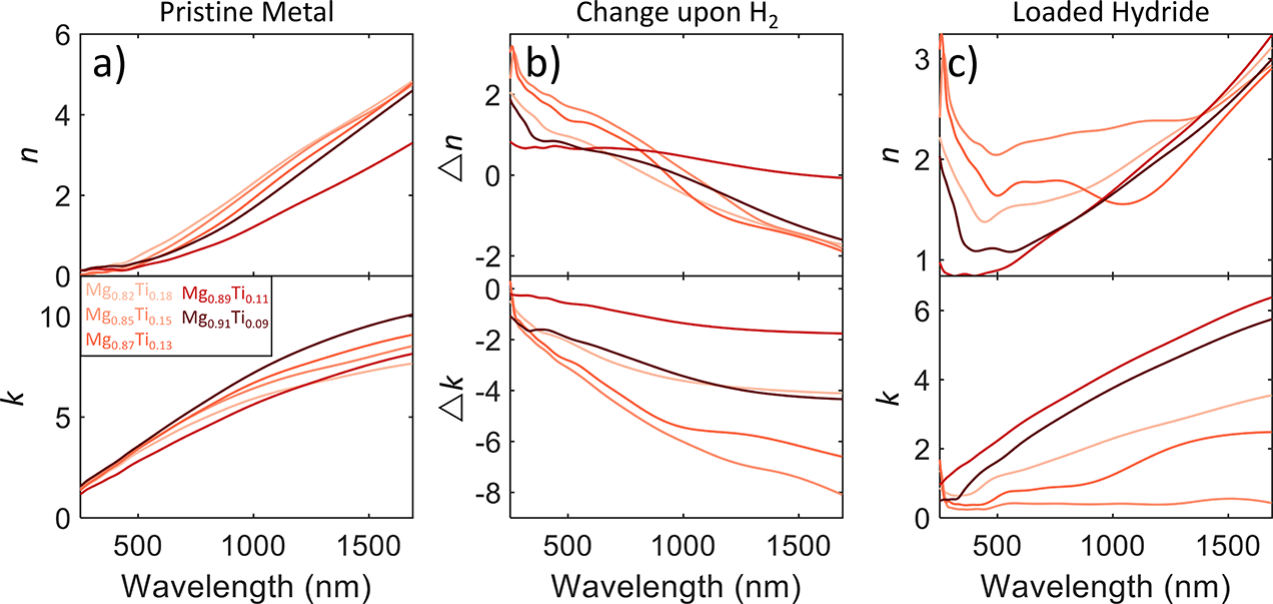
Optical properties *ñ* = *n* + i*k* of five different Mg–Ti alloy hydrides. (a) Optical properties in the pristine metallic state before hydrogenation. (b) Change in optical properties upon hydrogenation, defined here as the pure metal optical properties subtracted from the hydride optical properties. (c) Optical properties in the fully hydrogenated hydride state. Each colored line on the plot represents a different atomic ratio of metal hydride, with darker shades representing higher atomic Mg percent ratios.

Figure 3a shows the metallic optical properties of five different fabricated Mg–Ti alloys with the atomic Mg percent ranging from 82–91%. We find that the *n* values in the metallic state are fairly close together, with the exception of Mg_0.89_Ti_0.11_, which exhibits a lower *n* for the state. The higher Mg percent alloys exhibit higher *k* values, again except for Mg_0.89_Ti_0.11_ (we will discuss the discrepancies of the Mg_0.89_Ti_0.11_ sample further down). Higher attenuation for samples with more Mg is expected as Mg has much higher *k* values than Ti in their unalloyed form. The metallic properties of these Mg–Ti samples also align fairly closely with the metallic Mg–Al alloys investigated in the previous section. As we hydrogenate these samples, we find similar types of changes in the optical properties when compared to the Mg–Al samples, with *n* increasing in the visible and decreasing the in the NIR spectrum, and with *k* decreasing for all wavelengths, with larger decreases for longer wavelengths. There is a much broader range in the changes in properties for these alloys, and two alloys, Mg_0.85_Ti_0.15_ and Mg_0.87_Ti_0.13_, exhibit larger changes than the Mg–Al samples. Figure 3c shows the properties of the hydride states. The three lowest Mg percent samples show somewhat constant *n* in the visible spectrum, with peaks in the ultraviolet, and increasing values for longer wavelengths into the NIR spectrum. The two highest Mg percent samples have smaller *n* values in the visible, with sharper minima that monotonically increase with longer wavelengths, similar to the Mg–Al hydrides. These two samples also have the largest *k* by a significant margin. The lower Mg percent samples have relatively smaller *k* values, with Mg_0.85_Ti_0.15_ exhibiting almost no attenuation across the visible and NIR spectrum.

**Figure 4:**
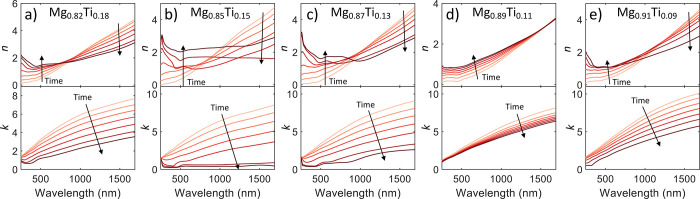
Optical properties of different Mg–Ti alloys as they are exposed to H_2_ gas. The lightest colored line depicts the alloy in the pristine metallic state. As H_2_ is introduced to the system, the material begins to hydrogenate, denoted by the lines getting darker in the plot, with the darkest line indicating the full hydride state. Each line is not linearly spaced in time and is instead chosen to aesthetically show the range of possible intermediate states. The alloys shown here are (a) Mg_0.82_Ti_0.18_, (b) Mg_0.85_Ti_0.15_, (c) Mg_0.87_Ti_0.13_, (d) Mg_0.89_Ti_0.11_, and (e) Mg_0.91_Ti_0.09_.

As shown in Figure 4, we observe the intermediate states during the hydrogen loading. These plots mostly show smooth optical transitions, except for Mg_0.85_Ti_0.15_ and Mg_0.87_Ti_0.13_, which show a resonance-like dip in *n* between 500 and 600 nm for high hydrogen content states. The Mg_0.82_Ti_0.18_ sample also slightly shows this effect. For the Mg_0.85_Ti_0.15_ sample, we also observe a decrease in *n* in the NIR spectrum until the almost fully hydrogenated state and then a sharp increase in *n* when the hydrogenation is complete. Note that again the chosen curves on this plot are not linearly spaced with time but are instead shown to depict the range of properties of the intermediate states.

#### 3.1.3 Mg–Ni Hydrides

**Figure 5:**
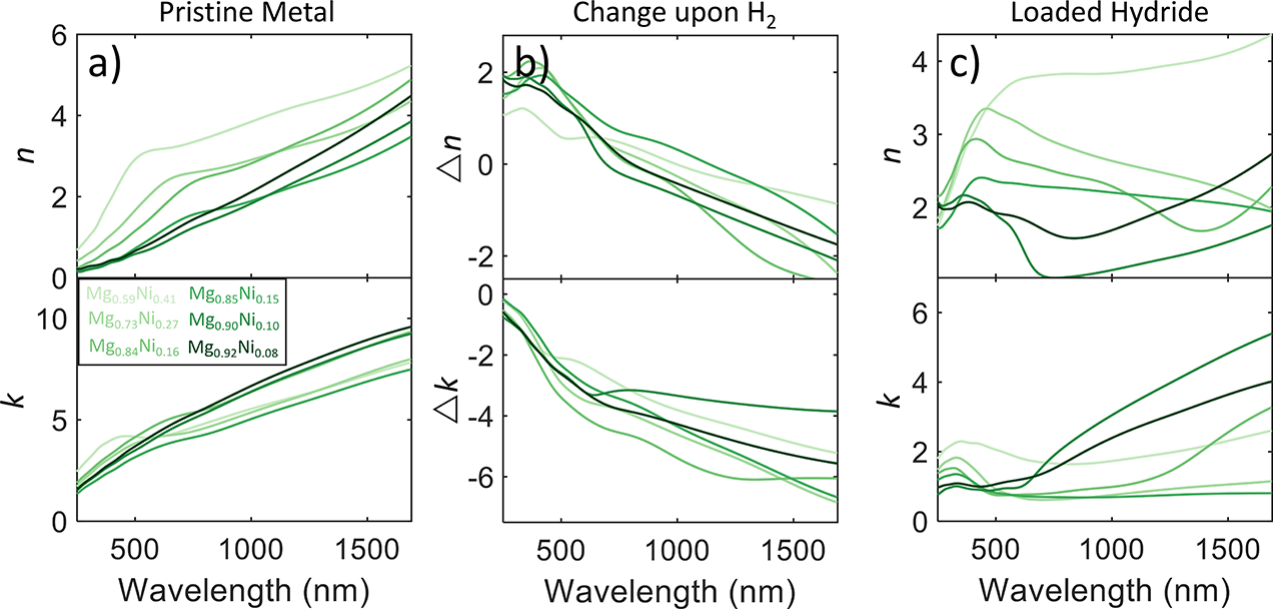
Optical properties *ñ* = *n* + i*k* of six different Mg–Ni alloy hydrides. (a) Optical properties in the pristine metallic state before hydrogenation. (b) Change in optical properties upon hydrogenation, defined here as the pure metal optical properties subtracted from the hydride optical properties. (c) Optical properties in the fully hydrogenated hydride state. Each colored line on the plot represents a different atomic ratio of metal hydride, with darker shades representing higher atomic Mg percent ratios.

Lastly, we investigate the properties of Mg–Ni alloys. Figure 5a shows the properties in the metallic state, where we see a much larger spread in initial *n* values for these materials, but *n* and *k* still follow the same trend as was found with the other Mg alloys (*n* and *k* generally increase with increasing wavelength). This larger spread in initial properties is expected as we are able to obtain a larger range of atomic ratios for this alloy system compared to the other two, ranging from 59–92% Mg. In Figure 5b, we see the same trends of optical property change as Mg–Al and Mg–Ti with large decreases in *k* across the spectrum upon hydrogenation and increases in *n* in the visible spectrum with decreases in the NIR spectrum. In the hydride state in Figure 5c, we see a large range of potential final optical properties depending upon the alloy ratio. Generally, the lower Mg percent hydrides have a higher *n* across the spectrum, with the Mg_0.90_Ni_0.10_ sample demonstrating the lowest *n* across most of the spectrum and Mg_0.59_Ni_0.41_ the highest. Most of the hydrides have low attenuation in the visible spectrum, with *k* < 2. The higher Mg percent hydrides then have larger attenuation into the NIR spectrum, while the lower Mg percent samples exhibit a more constant *k* across the measured spectrum.

**Figure 6:**
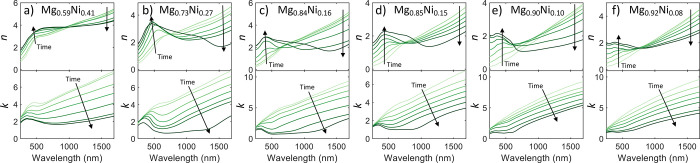
Optical properties of different Mg–Ni alloys as they are exposed to H_2_ gas. The lightest colored line depicts the alloy in the pristine metallic state. As H_2_ is introduced to the system, the material begins to hydrogenate, denoted by the lines getting darker in the plot, with the darkest line indicating the full hydride state. Each line is not linearly spaced in time and is instead chosen to aesthetically show the range of possible intermediate states. The alloys shown here are (a) Mg_0.59_Ni_0.41_, (b) Mg_0.73_Ni_0.27_, (c) Mg_0.84_Ni_0.16_, (d) Mg_0.85_Ni_0.15_, (e) Mg_0.90_Ni_0.10_, and (f) Mg_0.92_Ni_0.08_.

Figure 6 shows the dynamic transition-state data for the Mg–Ni samples. For each of the samples, the transitions are mostly monotonic without any exotic features. For the intermediate loading states, here we are modeling the Mg–Ni film as homogeneous throughout the thickness of the film. As was discussed in the Introduction section, it has been found that Mg–Ni films do not load homogeneously but instead with preferential phase formation from the substrate of the film that propagates to the Pd cap. Our modeling process is not contradictory to this process and still allows for the bulk characterization of the properties of the film in this orientation with illumination through the Pd cap. The low mean squared error (MSE) obtained in our optical fits indicates that our model accurately captures the optical properties of our samples. For our dynamic fits, the MSE for any individual fit was never greater than 10, indicating a good fit. However, for a different illumination orientation of the device (i.e., backside illumination through a transparent substrate), our intermediate model fits would not account for the layering effects of the loading. To model those bulk responses with our setup, the optical properties would have to be measured in that same orientation. Note that this layering effect would only affect the properties in the intermediate states and have no effect on the modeling of the metallic or fully hydrogenated states, as shown in Figure 5.

**Figure 7:**
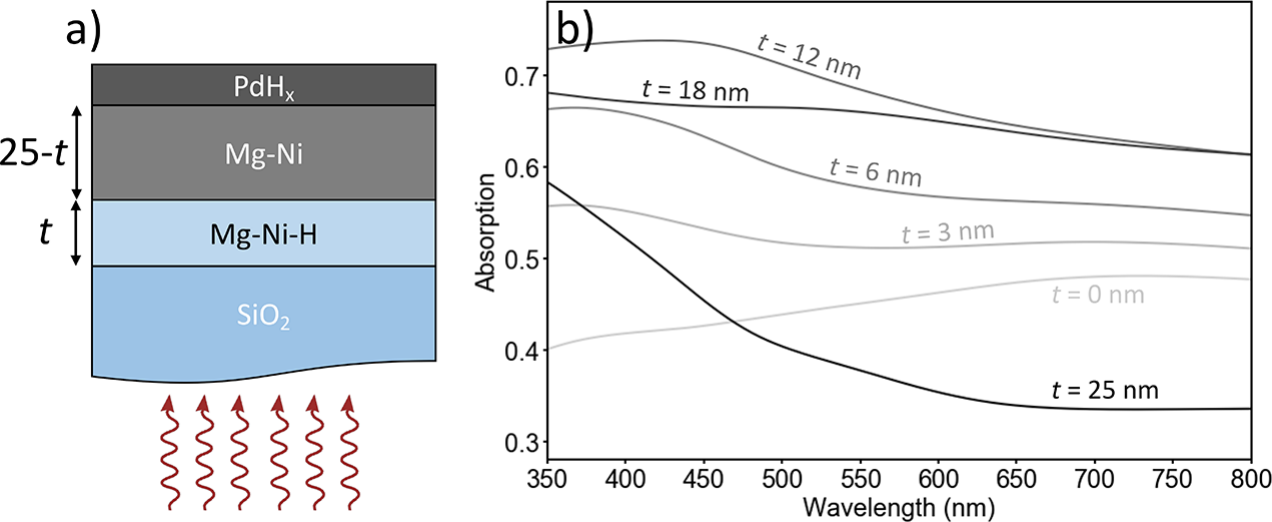
Modeled absorption from backside illumination of Mg_0.73_Ni_0.27_. (a) Simulation architecture for the sample loading. Sample consists of a SiO_2_ substrate, followed by a fully hydrogenated Mg–Ni–H layer, then a fully metallic Mg–Ni layer, and finally a 3 nm Pd capping layer. The total Mg alloy thickness is defined to be 25 nm. Simulated hydrogenation is defined as an increase in the thickness of the hydride layer and a decrease of the same magnitude of the metal layer. (b) Calculated absorption for different loading thicknesses using Mg_0.73_Ni_0.27_ optical properties.

To determine whether any of our materials would exhibit this black state with backside illumination, we modeled the multi-layer loading process with TMM simulations. Figure 7a shows the simulation architecture. The samples consist of illumination through a SiO_2_ substrate. The layers proceed from the substrate with the fully hydrogenated Mg–Ni hydride, the fully metallic Mg–Ni alloy, and lastly a 3 nm PdH_*x*_ capping layer. The hydrogenation of the material is simulated by beginning with the Mg–Ni–H layer equal to 0 nm and then increasing this layer size while decreasing the Mg–Ni layer by the same amount, until the sample is completely hydrogenated. We defined the alloy thickness to be 25 nm for these simulations. Using this method, we find a high-absorbing intermediate state for multiple samples, with the largest absorption occurring for the Mg_0.73_Ni_0.27_ sample, which is expected as this material is the closest composition to Mg_2_Ni for which the black state was initially discovered. In Figure 7b, we show the absorption curves for different loading thicknesses for this alloy, where the peak absorption occurs at a hydride thickness of 12 nm, which is equal to half of the thin film being loaded.

### 3.2 Stress and Loading Properties

**Figure 8:**
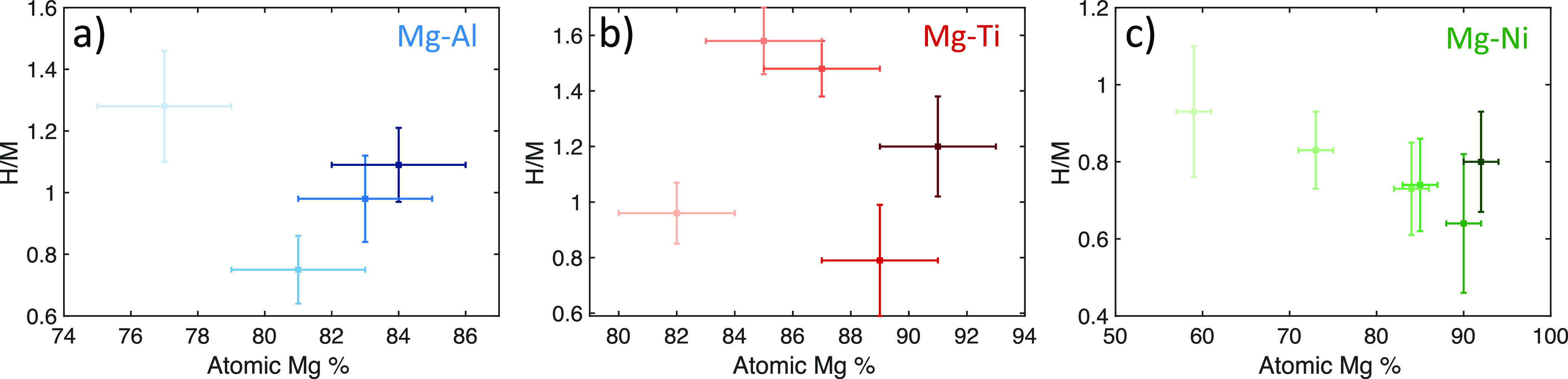
Measured maximum loading values under 1 bar H_2_ for different thin films for (a) Mg–Al, (b) Mg–Ti, and (c) Mg–Ni alloy samples. The horizontal and vertical error bars represent one standard deviation uncertainty in the atomic Mg % and the loading, respectively.

With the second QCM crystal included in the deposition chamber, we dynamically measure the loading and stress properties of the films. The loading values for all the samples are shown in Figure 8. Figure 8a shows the loading values of the Mg–Al samples. We can see that the samples have loadings near H/M = 1, with the highest measured loading at 1.28 for Mg_0.77_Al_0.23_ and the lowest loading at 0.75 for Mg_0.81_Al_0.19_. These values are at the low end of the range of hydrogen loading measurements reported in the literature for alloys in this composition range, which find loading values between H/M = 0.85–1.5.^22,44^ The Mg_0.77_Al_0.23_ also exhibited the largest optical property change compared to the other Mg–Al samples. Future work looking at these samples should investigate fabrication of higher Al atomic percentages to determine if there is a correlation of higher loading for higher Al percent for any range of compositions and if the optical property change is greater in this region.

The loading values of the Mg–Ti films are shown in Figure 8b. Three of these values are in general agreement in the literature, which finds that the average amount of loading of Mg–Ti alloys in this atomic composition range is H/M = 1.55.^26,41^ We find two samples that fall measurably below this average, with Mg_0.82_Ti_0.18_ at 0.96 and Mg_0.89_Ti_0.11_ at 0.79. We also find that the Mg_0.89_Ti_0.11_ sample had the lowest optical change of any of the investigated alloys. We suspect that there was an issue with the fabrication of this sample that prevented a complete loading. This could have been caused by an incomplete Pd capping layer that did not fully encapsulate the sample, allowing for oxidation of the surface of the alloy, or potential alloying between the Pd capping layer and Mg near the surface of the Mg–Ti alloy.

For Mg–Ni alloys in Figure 8c, we see generally lower calculated loadings than that for the other two alloys. We also find a slightly negative correlation between the loading amount and the atomic Mg percent. This does not agree with previously found data in the literature, which found that there should be a slightly positive correlation between these values and that they should fall between 1.2 and 1.4 H/M.^31^ This loading difference could be attributed to differences in sample preparation. Other thin-film Mg–Ni alloys have been fabricated with multi-layer metal deposition followed by a high-temperature anneal, as opposed to our cosputtering method. These different fabrication conditions could potentially be forming different alloying phases within the metal, which would affect the total loading amount. Further studies on the crystal structures of Mg–Ni alloys fabricated with these two techniques should be carried out to determine if there is any difference.

**Figure 9:**
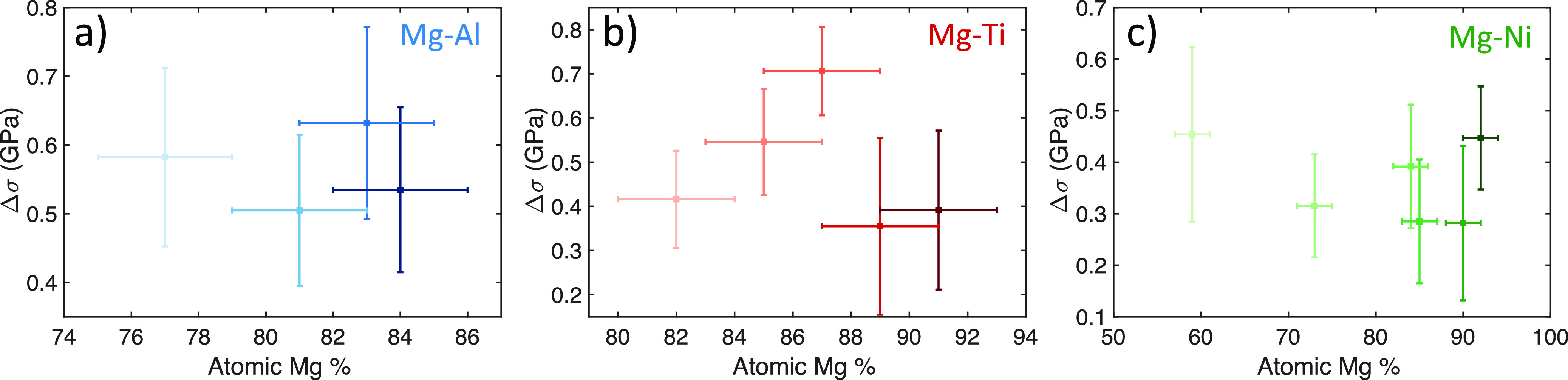
Measured total stress change values for different thin-film samples for (a) Mg–Al, (b) Mg–Ti, and (c) Mg–Ni alloy samples. The change in stress reported here is the stress change from the initial pristine metal mounted in the environmental chamber to the fully hydrogenated state under 1 bar H_2_ (does not include intrinsic stress of the initial pristine alloy). The horizontal and vertical error bars represent one standard deviation uncertainty in the atomic Mg % and the stress change, respectively.

In Figure 9, we show the total stress change of each of the measured Mg alloys upon full hydrogenation. These stress changes are compressive and are defined to be positive. We find the stresses for the samples to be consistent within a material system. Taking the averages of the stresses in each system, the Mg–Al samples have the highest stresses with an average of 0.56 GPa, next is Mg–Ti with an average of 0.48 GPa, and finally the Mg–Ni alloys have the lowest measured stress with an average of 0.36 GPa.

### 3.3 Applications

**Figure 10:**
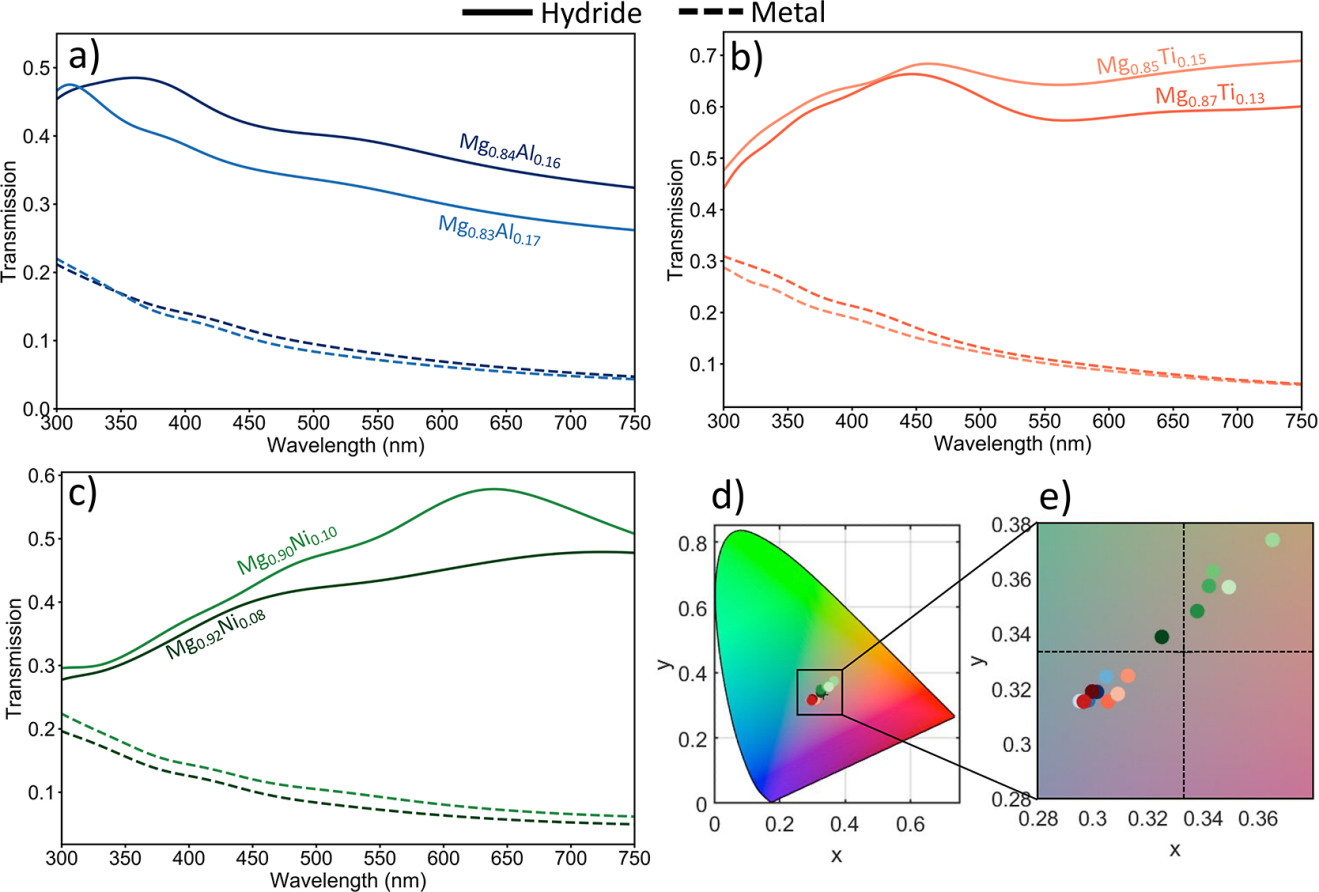
Switchable windows with Mg alloys. Simulated transmission values of thin films (a) Mg–Al, (b) Mg–Ti, and (c) Mg–Ni alloys on SiO_2_. The stack is defined as an SiO_2_ substrate, a 25 nm Mg alloy film, and a 3 nm Pd capping layer. Transmission in the hydride (metal) state is represented by solid (dashed) lines. (d) CIE 1931 chromaticity diagram with the transmission color points for the different alloys. Colors of the points match with the hues in part a–c. (e) Zoom-in of chromaticity diagram. The intersection of the black dashed lines represents color neutral at *x* = *y* = 0.333.

In this section, we use our measured optical properties of the alloys to demonstrate three potential applications. We use the TMM to simulate thin-film responses of these materials for different thicknesses and on distinct substrates. The first application of these materials is for switchable window technologies. As an example, we simulate the transmission through the alloys in their metallic and hydride states and compare the transmission amounts. We use a metallic alloy thickness of 25 nm with a 3 nm Pd capping layer. We find that these thicknesses are in the ideal range for switchable window purposes because it is just thick enough in the metallic state to create high reflection, while remaining thin enough to allow appreciable transmission in the hydride state. These simulations also consider the 15% volume expansion of the alloys upon hydrogenation. Another important factor in window technologies is to have color-neutral transmission. Windows with non-neutral color transmission tint the light as it transmits through the window, which is not ideal when attempting to make a clear window. To model this color distortion, we use the CIE 1931 XYZ color space and plot the perceived colors of the transmitted spectrum. Note that only *x* and *y* need to be plotted to fully characterize the color because *x* + *y* + *z* = 1. On these plots, color-neutral is the *x* = *y* = 0.333 data point, which creates the ideal window. We show these switchable window properties for all the investigated alloys in Figure 10.

For the Mg–Al alloys, we see poor transmission in the hydride state, with the Mg_0.84_Al_0.16_ alloy having the highest transmission through the visible spectrum with values <40% transmission for most of the spectrum. These materials generally only exhibit ∼20% absolute change in transmission upon hydrogenation, agreeing with results previously reported in the literature for Mg_*x*_Al_1–*x*_ with *x* > 0.69.^21^ This behavior is due to the materials still exhibiting a high attenuation in the visible, even in the hydride state. These windows are close to color neutral, even with their low transmission, adding a small blue–green tint to the transmitted light.

Some of the Mg–Ti alloys perform much better as switchable window technologies with transmissions values >60% across most of the visible spectrum for Mg_0.85_Ti_0.15_ and Mg_0.87_Ti_0.13_. For these samples, we observe a transmission change of ∼40%. Higher transmission can be achieved in the hydride state by a thinning of the sample; however, this thinning causes the transmission in the metallic state to also become significantly higher. We find the transmission colors of these windows to be similar to those of the Mg–Al samples, being mostly color neutral with a slight blue–green tint. Mg_0.85_Ti_0.15_ is the most color neutral, in addition to having the highest transmission across most of the visible spectrum.

The Mg–Ni alloys measured in this manuscript have poor switchable window characteristics, with low transmissions in the hydride state in the shorter wavelength visible region, with similar results for a Mg-rich Mg–Ni films having been found previously where a peak transmission of 25% was found for an 80 nm thin film.^32^ For the longer wavelength visible, we see increased transmission but still only achieve values of ∼50%. However, we do see ∼40% transmission changes in this region. Some Mg–Ni samples exhibit very good color neutrality, with Mg_0.92_Ni_0.08_ having a transmission color value of *x* = 0.325 and *y* = 0.338. The other alloys have a slight yellow tint, as opposed to the blue–green tint of the Mg–Al and Mg–Ti alloys.

**Figure 11:**
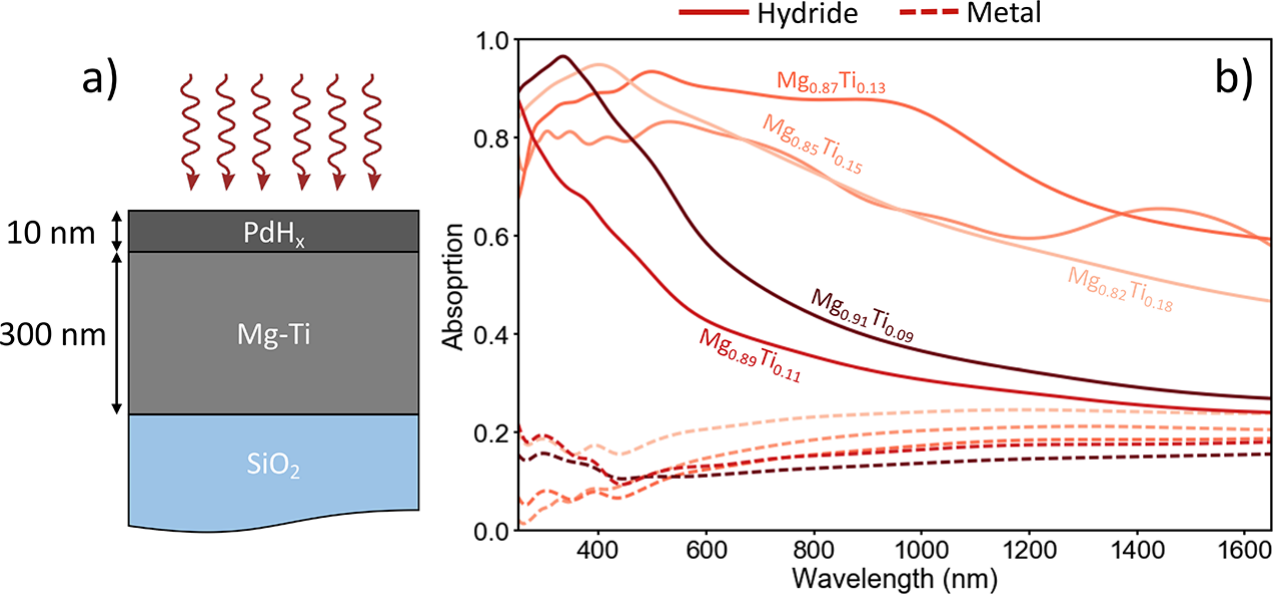
Broadband switchable light absorbers using Mg–Ti alloys. (a) Schematic of switchable light absorber consisting of a 300 nm Mg–Ti alloy with a 10 nm Pd cap on a SiO_2_ substrate. (b) Absorption plots for this structure for different alloy compositions. Solid (dashed) lines are absorption in the hydride (metallic) state. Colors represents different alloy compositions.

As mentioned in the Introduction, Mg–Ti alloys have also been investigated as broadband switchable light absorbers, with a highly reflecting state in the metallic form and a highly absorbing state when hydrogenated. The high absorption states for these materials have been found with thicker samples >200 nm, much thicker than measured here, and it has been demonstrated that the total absorption of the film can be significantly tuned by varying the thickness of the film.^26,49^ To see if our measured properties show any potential for switchable absorption, we modeled a 300 nm Mg–Ti film on an SiO_2_ substrate with a 10 nm Pd capping layer. The results of these simulations are shown in Figure 11. We see that for three of our measured Mg–Ti alloys, we achieve large amounts of switchable absorption throughout the visible spectrum, with absorption tailing off into the NIR spectrum. In the visible wavelength region, Mg_0.87_Ti_0.13_, Mg_0.85_Ti_0.15_, and Mg_0.82_Ti_0.18_ all achieve >80% absorption in the hydride state with <25% absorption in the metallic state (corresponding to high reflection in this state). This is a very large absorption change upon hydrogenation for these alloy compositions and shows their potential for broadband switchable light absorbers. These results agree with the previously reported literature showing that Mg_*x*_Ti_1–*x*_ alloys with *x* ≈ 0.2 is an excellent chemical composition for solar absorption.^25^

**Figure 12:**
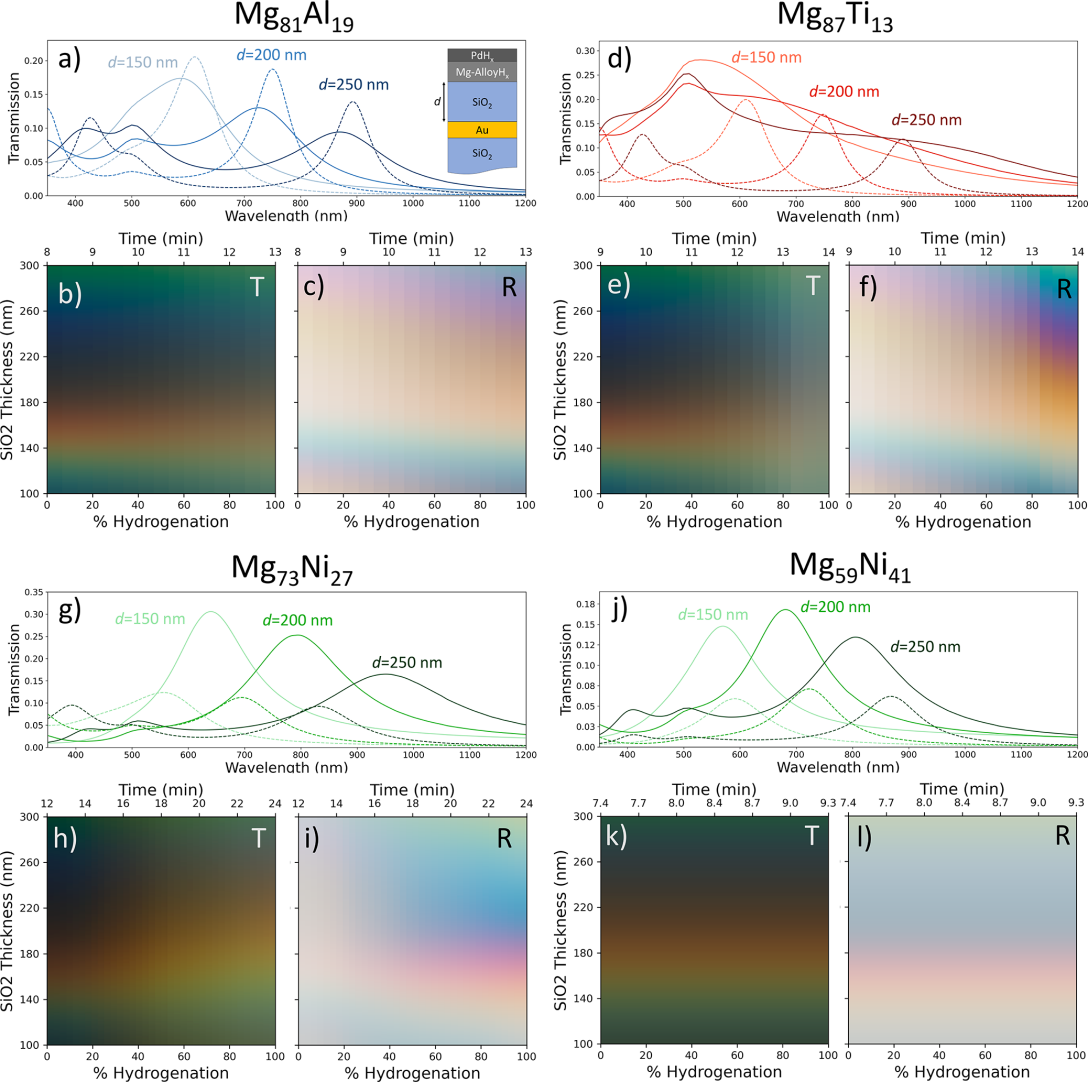
Dual-functionality reflective and transmissive color filters with Mg alloy hydrides. (a) Simulated transmission plots of the Mg_81_Al_19_ color filter in both the metallic and hydride states with 150, 200, and 250 nm SiO_2_ cavity thickness. The inset shows the schematic of the proposed tunable color filter structure and defines the cavity thickness *d*. (b) Reflected and (c) transmitted colors of the device at different hydrogenation levels and cavity thicknesses. Similar plots are shown for (d–f) Mg_87_Ti_13_, (g–i) Mg_73_Ni_27_, and (j–l) Mg_59_Ni_41_.

Finally, we investigate the potential of utilizing these metal alloys as tunable color filters. Our proposed structure uses a cavity structure, consisting of a 20 nm Au backplane on SiO_2_, a variable length SiO_2_ cavity, capped with 25 nm of Mg alloy with a 3 nm Pd cap. We find that the Mg–Ti alloys have the most vibrant color change, with Mg_87_Ti_17_ exhibiting a change from a pale orange to a vibrant blue with an SiO_2_ cavity length of 260 nm. The Mg–Ni alloys showed an interesting effect upon hydrogenation, with some alloys exhibiting a blue-shift in color change upon hydrogenation, while other exhibit a red-shift (Figure 12g–l). Combining these different alloys into a single structure could allow for an even greater relative color change in a device. The Mg–Al shows the smallest color change of the alloys investigated in either the reflection or transmission spectrum.

## 4 Conclusions

In conclusion, we have for the first time experimentally measured the complex optical properties of different compositions of Mg–Al, Mg–Ti, and Mg–Ni alloys during the hydrogenation process using variable-angle spectroscopic ellipsometry. By quantifying changes in the refractive index of these alloys, we accurately designed three classes of devices: switchable windows, solar absorbers, and color filters. Our results show the chemical composition dependence on the optical properties of each of these devices, agreeing with the literature on overlapping compositional data points for the switchable windows and solar absorbers, with no literature suggesting tunable color filters with these materials to the authors’ knowledge. Surprisingly, we found a wide range in optical properties for the alloys in the final hydrogenated state, albeit most of the samples showing similar properties in the metallic state. We dynamically measured the loadings and stresses of all samples and found that the loadings of our materials are less than those previously reported in the literature, suggesting that these material properties are likely sensitive to fabrication differences. Future work will focus on elucidating the effects of different fabrication techniques (evaporation vs sputtering, annealing affects, substrate preparation, etc.) on the different material properties of these alloys. We also explored the implementation of these materials into optical devices, showing that Mg_0.87_Ti_0.13_ and Mg_0.85_Ti_0.15_ can achieve 40% transmission changes as switchable windows and 55% absorption changes as switchable solar absorbers. The alloys could also be used as active layers in tunable color filters. The switching process of the dual-reflective-transmissive filter introduced here is scalable and reveals a previously overlooked class of materials, Mg-based-alloys, as a model system for color pixels to be applied in situations where reconfigurability is desired, for example, encryption, tunable windows, or holography.

## References

[ref1] Palm K. J.; Murray J. B.; Narayan T. C.; Munday J. N. Dynamic Optical Properties of Metal Hydrides. ACS Photonics 2018, 5, 4677–4686. 10.1021/acsphotonics.8b01243.

[ref2] Palm K. J.; Murray J. B.; McClure J. P.; Leite M. S.; Munday J. N. In Situ Optical and Stress Characterization of Alloyed Pd_*x*_Au_1-*x*_ Hydrides. ACS Appl. Mater. Interfaces 2019, 11, 45057–45067. 10.1021/acsami.9b14244. 31670929

[ref3] Gong T.; Lyu P.; Palm K. J.; Memarzadeh S.; Munday J. N.; Leite M. S. Emergent Opportunities with Metallic Alloys: From Material Design to Optical Devices. Adv. Opt. Mater. 2020, 8, 2001082 10.1002/adom.202001082.

[ref4] Duan X.; Kamin S.; Liu N. Dynamic Plasmonic Colour Display. Nat. Commun. 2017, 8, 1–9. 10.1038/ncomms14606. 28232722 PMC5333121

[ref5] Bagheri S.; Strohfeldt N.; Ubl M.; Berrier A.; Merker M.; Richter G.; Siegel M.; Giessen H. Niobium as Alternative Material for Refractory and Active Plasmonics. ACS Photonics 2018, 5, 3298–3304. 10.1021/acsphotonics.8b00530.

[ref6] Wadell C.; Syrenova S.; Langhammer C. Plasmonic Hydrogen Sensing with Nanostructured Metal Hydrides. ACS Nano 2014, 8, 11925–11940. 10.1021/nn505804f. 25427244

[ref7] Baldi A.; Borsa D. M.; Schreuders H.; Rector J. H.; Atmakidis T.; Bakker M.; Zondag H. A.; van Helden W. G. J.; Dam B.; Griessen R. Mg-Ti-H Thin Films as Switchable Solar Absorbers. Int. J. Hydrogen Energy 2008, 33, 3188–3192. 10.1016/j.ijhydene.2008.01.026.

[ref8] Darmadi I.; Khairunnisa S. Z.; Tomeček D.; Langhammer C. Optimization of the Composition of PdAuCu Ternary Alloy Nanoparticles for Plasmonic Hydrogen Sensing. ACS Appl. Nano Mater. 2021, 4, 8716–8722. 10.1021/acsanm.1c01242.

[ref9] Stampfer J. F.; Holley C. E.; Suttle J. F. The Magnesium-Hydrogen System^1-3^. J. Am. Chem. Soc. 1960, 82, 3504–3508. 10.1021/ja01499a006.

[ref10] Stander C. M. Kinetics of Decomposition of Magnesium Hydride. J. Inorg. Nucl. Chem. 1977, 39, 221–223. 10.1016/0022-1902(77)80003-1.

[ref11] Reiser A.; Bogdanović B.; Schlichte K. The Application of Mg-Based Metal-Hydrides as Heat Energy Storage Systems. Int. J. Hydrogen Energy 2000, 25, 425–430. 10.1016/S0360-3199(99)00057-9.

[ref12] González Fernández I.; Gennari F. C.; Meyer G. O. Influence of Sintering Parameters on Formation of Mg-Co Hydrides Based on Their Thermodynamic Characterization. J. Alloys Compd. 2008, 462, 119–124. 10.1016/j.jallcom.2007.08.038.

[ref13] Shao H.; Liu T.; Wang Y.; Xu H.; Li X. Preparation of Mg-Based Hydrogen Storage Materials from Metal Nanoparticles. J. Alloys Compd. 2008, 465, 527–533. 10.1016/j.jallcom.2007.11.003.

[ref14] Richardson T. J.; Slack J. L.; Farangis B.; Rubin M. D. Mixed Metal Films with Switchable Optical Properties. Appl. Phys. Lett. 2002, 80, 1349–1351. 10.1063/1.1454218.

[ref15] Lima G. F.; Jorge A. M.; Leiva D. R.; Kiminami C. S.; Bolfarini C.; Botta W. J. Severe Plastic Deformation of Mg-Fe Powders to Produce Bulk Hydrides. J. Phys. Conf. 2009, 144, 012015 10.1088/1742-6596/144/1/012015.

[ref16] Berlouis L. E. A.; Cabrera E.; Hall-Barientos E.; Hall P. J.; Dodd S. B.; Morris S.; Imam M. A. Thermal Analysis Investigation of Hydriding Properties of Nanocrystalline Mg-Ni- and Mg-Fe-Based Alloys Prepared by High-Energy Ball Milling. J. Mater. Res. 2001, 16, 45–57. 10.1557/JMR.2001.0012.

[ref17] Lu Y.; Kim H.; Sakaki K.; Hayashi S.; Jimura K.; Asano K. Destabilizing the Dehydrogenation Thermodynamics of Magnesium Hydride by Utilizing the Immiscibility of Mn with Mg. Inorg. Chem. 2019, 58, 14600–14607. 10.1021/acs.inorgchem.9b02253. 31647662

[ref18] Gremaud R.; Borgschulte A.; Lohstroh W.; Schreuders H.; Züttel A.; Dam B.; Griessen R. Ti-Catalyzed Mg(AlH_4_)_2_: A Reversible Hydrogen Storage Material. J. Alloys Compd. 2005, 404–406, 775–778. 10.1016/j.jallcom.2005.01.140.

[ref19] Zaluska A.; Zaluski L.; Ström-Olsen J. O. Structure, Catalysis and Atomic Reactions on the Nano-Scale: A Systematic Approach to Metal Hydrides for Hydrogen Storage. Appl. Phys. A 2001, 72, 157–165. 10.1007/s003390100783.

[ref20] Bouaricha S.; Dodelet J. P.; Guay D.; Huot J.; Boily S.; Schulz R. Hydriding Behavior of Mg-Al and Leached Mg-Al Compounds Prepared by High-Energy Ball-Milling. J. Alloys Compd. 2000, 297, 282–293. 10.1016/S0925-8388(99)00612-X.

[ref21] Gremaud R.; Borgschulte A.; Chacon C.; van Mechelen J. L. M.; Schreuders H.; Züttel A.; Hjörvarsson B.; Dam B.; Griessen R. Structural and Optical Properties of Mg_*x*_Al_1–*x*_H_*y*_ Gradient Thin Films: A Combinatorial Approach. Appl. Phys. A 2006, 84, 77–85. 10.1007/s00339-006-3579-z.

[ref22] Fritzsche H.; Saoudi M.; Haagsma J.; Ophus C.; Luber E.; Harrower C. T.; Mitlin D. Neutron Reflectometry Study of Hydrogen Desorption in Destabilized MgAl Alloy Thin Films. Appl. Phys. Lett. 2008, 92, 121917 10.1063/1.2899936.

[ref23] Vermeulen P.; Graat P. C. J.; Wondergem H. J.; Notten P. H. L. Crystal structures of Mg_*y*_Ti_100–*y*_ thin film alloys in the as-deposited and hydrogenated state. Int. J. Hydrogen Energy 2008, 33, 5646–5650. 10.1016/j.ijhydene.2008.07.014.

[ref24] Kim H.; Schreuders H.; Sakaki K.; Asano K.; Nakamura Y.; Maejima N.; Machida A.; Watanuki T.; Dam B. Unveiling Nanoscale Compositional and Structural Heterogeneities of Highly Textured Mg_0.7_Ti_0.3_H_*y*_ Thin Films. Inorg. Chem. 2020, 59, 6800–6807. 10.1021/acs.inorgchem.0c00059. 32379436

[ref25] Borsa D. M.; Baldi A.; Pasturel M.; Schreuders H.; Dam B.; Griessen R.; Vermeulen P.; Notten P. H. L. Mg-Ti-H thin films for smart solar collectors. Appl. Phys. Lett. 2006, 88, 241910 10.1063/1.2212287.

[ref26] Borsa D. M.; Gremaud R.; Baldi A.; Schreuders H.; Rector J. H.; Kooi B.; Vermeulen P.; Notten P. H. L.; Dam B.; Griessen R. Structural, optical, and electrical properties of Mg_*y*_Ti_1–*y*_H_*x*_ thin films. Phys. Rev. B: Condens. Matter Mater. Phys. 2007, 75, 205408 10.1103/PhysRevB.75.205408.

[ref27] Niessen R. a. H.; Notten P. H. L. Electrochemical Hydrogen Storage Characteristics of Thin Film MgX (X = Sc, Ti, V, Cr) Compounds. Electrochem. Solid-State Lett. 2005, 8, A534 10.1149/1.2012238.

[ref28] Westerwaal R. J.; Borgschulte A.; Lohstroh W.; Dam B.; Kooi B.; ten Brink G.; Hopstaken M. J. P.; Notten P. H. L. The Growth-Induced Microstructural Origin of the Optical Black State of Mg_2_NiH_*x*_ Thin Films. J. Alloys Compd. 2006, 416, 2–10. 10.1016/j.jallcom.2005.07.068.

[ref29] Orimo S.; Fujii H. Materials Science of Mg-Ni-Based New Hydrides. Appl. Phys. A 2001, 72, 167–186. 10.1007/s003390100771.

[ref30] Ludwig A.; Cao J.; Dam B.; Gremaud R. Opto-mechanical characterization of hydrogen storage properties of Mg-Ni thin film composition spreads. Appl. Surf. Sci. 2007, 254, 682–686. 10.1016/j.apsusc.2007.05.093.

[ref31] Johansson E.; Chacon C.; Zlotea C.; Andersson Y.; Hjörvarsson B. Hydrogen Uptake and Optical Properties of Sputtered Mg-Ni Thin Films. J. Phys. Condens. Matter 2004, 16, 7649–7662. 10.1088/0953-8984/16/43/008.

[ref32] Richardson T. J.; Slack J. L.; Armitage R. D.; Kostecki R.; Farangis B.; Rubin M. D. Switchable mirrors based on nickel-magnesium films. Appl. Phys. Lett. 2001, 78, 3047–3049. 10.1063/1.1371959.

[ref33] Isidorsson J.; Giebels I. a. M. E.; Griessen R.; Di Vece M. Tunable Reflectance Mg-Ni-H Films. Appl. Phys. Lett. 2002, 80, 2305–2307. 10.1063/1.1463205.

[ref34] Lohstroh W.; Westerwaal R. J.; Noheda B.; Enache S.; Giebels I. A. M. E.; Dam B.; Griessen R. Self-Organized Layered Hydrogenation in Black Mg_2_NiH_*x*_ Switchable Mirrors. Phys. Rev. Lett. 2004, 93, 197404 10.1103/PhysRevLett.93.197404. 15600879

[ref35] Lohstroh W.; Westerwaal R. J.; van Mechelen J. L. M.; Chacon C.; Johansson E.; Dam B.; Griessen R. Structural and optical properties of Mg_2_NiH_*x*_ switchable mirrors upon hydrogen loading. Phys. Rev. B: Condens. Matter Mater. Phys. 2004, 70, 165411 10.1103/PhysRevB.70.165411.

[ref36] Gremaud R.; Broedersz C. P.; Borgschulte A.; van Setten M. J.; Schreuders H.; Slaman M.; Dam B.; Griessen R. Hydrogenography of Mg_*y*_Ni_1–*y*_H_*x*_ gradient thin films: Interplay between the thermodynamics and kinetics of hydrogenation. Acta Mater. 2010, 58, 658–668. 10.1016/j.actamat.2009.09.044.

[ref37] Pasturel M.; Slaman M.; Borsa D. M.; Schreuders H.; Dam B.; Griessen R.; Lohstroh W.; Borgschulte A. Stabilized Switchable Black State in Mg_2_NiH_4_/Ti/Pd Thin Films for Optical Hydrogen Sensing. Appl. Phys. Lett. 2006, 89, 021913 10.1063/1.2221412.

[ref38] Pivak Y.; Palmisano V.; Schreuders H.; Dam B. The Clamping Effect in the Complex Hydride Mg_2_NiH_4_ Thin Films. J. Mater. Chem. A 2013, 1, 10972–10978. 10.1039/C3TA11937H.

[ref39] Akiba E.; Nomura K.; Ono S.; Suda S. Kinetics of the Reaction between Mg-Ni Alloys and H_2_. Int. J. Hydrogen Energy 1982, 7, 787–791. 10.1016/0360-3199(82)90069-6.

[ref40] Zhou C.; Fang Z. Z.; Lu J.; Luo X.; Ren C.; Fan P.; Ren Y.; Zhang X. Thermodynamic Destabilization of Magnesium Hydride Using Mg-Based Solid Solution Alloys. J. Phys. Chem. C 2014, 118, 11526–11535. 10.1021/jp501306w.

[ref41] Vermeulen P.; Niessen R. A. H.; Notten P. H. L. Hydrogen storage in metastable Mg_*y*_Ti_(1–*y*)_ thin films. Electrochem. Commun. 2006, 8, 27–32. 10.1016/j.elecom.2005.10.013.

[ref42] Bao S.; Tajima K.; Yamada Y.; Okada M.; Yoshimura K. Magnesium-Titanium Alloy Thin-Film Switchable Mirrors. Sol. Energy Mater. Sol. Cells 2008, 92, 224–227. 10.1016/j.solmat.2007.02.024.

[ref43] Slaman M.; Dam B.; Schreuders H.; Griessen R. Optimization of Mg-Based Fiber Optic Hydrogen Detectors by Alloying the Catalyst. Int. J. Hydrogen Energy 2008, 33, 1084–1089. 10.1016/j.ijhydene.2007.09.036.

[ref44] Gremaud R.; van Mechelen J. L. M.; Schreuders H.; Slaman M.; Dam B.; Griessen R. Structural and Optical Properties of Mg_*y*_Ni_1-*y*_H_*x*_ Gradient Thin Films in Relation to the as-Deposited Metallic State. Int. J. Hydrogen Energy 2009, 34, 8951–8957. 10.1016/j.ijhydene.2009.08.051.

[ref45] Lohstroh W.; Westerwaal R. J.; van Mechelen J. L. M.; Schreuders H.; Dam B.; Griessen R. The Dielectric Function of Mg_*y*_NiH_*x*_ Thin Films (2<*y*<10). J. Alloys Compd. 2007, 430, 13–18. 10.1016/j.jallcom.2006.04.075.

[ref46] King R. J.; Talim S. P. A Comparison of Thin Film Measurement by Guided Waves, Ellipsometry and Reflectometry. Opt. Acta Int. J. Opt. 1981, 28, 1107–1123. 10.1080/713820674.

[ref47] Murray J. B.; Palm K. J.; Narayan T. C.; Fork D. K.; Sadat S.; Munday J. N. Apparatus for Combined Nanoscale Gravimetric, Stress, and Thermal Measurements. Rev. Sci. Instrum. 2018, 89, 085106 10.1063/1.5040503. 30184662

[ref48] Slaman M.; Dam B.; Pasturel M.; Borsa D. M.; Schreuders H.; Rector J. H.; Griessen R. Fiber Optic Hydrogen Detectors Containing Mg-Based Metal Hydrides. Sens. Actuators, B 2007, 123, 538–545. 10.1016/j.snb.2006.09.058.

[ref49] Farangis B.; Nachimuthu P.; Richardson T. J.; Slack J. L.; Meyer B. K.; Perera R. C. C.; Rubin M. D. Structural and Electronic Properties of Magnesium-3D Transition Metal Switchable Mirrors. Solid State Ionics 2003, 165, 309–314. 10.1016/j.ssi.2003.08.041.

